# CSF Neopterin Levels Are Elevated in Various Neurological Diseases and Aging

**DOI:** 10.3390/jcm13154542

**Published:** 2024-08-03

**Authors:** Noriyuki Miyaue, Yuki Yamanishi, Yuko Ito, Rino Ando, Masahiro Nagai

**Affiliations:** Department of Clinical Pharmacology and Therapeutics, Ehime University Graduate School of Medicine, Tohon 791-0295, Japan; yamanishi.yuki.vz@ehime-u.ac.jp (Y.Y.); ito.yuko.et@ehime-u.ac.jp (Y.I.); ando.rina.cn@ehime-u.ac.jp (R.A.); mnagai@m.ehime-u.ac.jp (M.N.)

**Keywords:** neopterin, cerebrospinal fluid, age, amyotrophic lateral sclerosis, inflammation

## Abstract

**Background/Objectives**: Cerebrospinal fluid (CSF) neopterin reflects inflammation of the central nervous system (CNS) and is a potentially useful biomarker for neuroinflammatory assessment and differential diagnosis. However, its optimal cut-off level in adult patients with neurological disease has not been established and it has not been adequately studied in controls. We aimed to determine its usefulness as a biomarker of neuroinflammation and the effect of age on its level. **Methods**: In this retrospective study, CSF neopterin was evaluated in 652 patients in 38 disease groups. Its levels were analyzed with high-performance liquid chromatography with fluorometric detection. **Results**: A receiver operating characteristic analysis revealed that the optimal cut-off value of 33.57 pmol/mL for CSF neopterin distinguished the control and meningitis/encephalitis groups with a sensitivity of 100.0% and specificity of 94.4%. In the control group, which consisted of 170 participants (99 men and 71 women; mean ± standard deviation age, 52.56 ± 17.99 years), age was significantly positively correlated with CSF protein (*r* = 0.474, *p* < 0.001) and CSF neopterin (*r* = 0.476, *p* < 0.001) levels but not with CSF cell count (*r* = 0.144, *p* = 0.061). Both male and female controls exhibited significant increases in CSF neopterin levels with age. Similarly, the CSF neopterin level was significantly positively correlated with age in patients with amyotrophic lateral sclerosis, independently of disease duration and respiratory function. **Conclusions**: CSF neopterin levels were elevated in patients with various CNS diseases, reflecting CNS inflammation; they were also elevated with age. Prospective studies are required to establish CSF neopterin as a sensitive biomarker of neuroinflammation.

## 1. Introduction

Neopterin is a pyrazino-pyrimidine compound that is synthesized from guanosine triphosphate by macrophages, monocytes, microglia, and dendritic cells in response to stimulation with the interferon-γ produced by activated T cells. The serum and cerebrospinal fluid (CSF) neopterin levels are not clearly correlated, and CSF neopterin appears to be produced in the central nervous system (CNS) [1]. Thus, CSF neopterin can serve as an indicator of cell-mediated immune responses in the CNS. Elevated levels of CSF neopterin have been reported in various CNS diseases, including CNS infections [2], autoimmune encephalitis [3], human T-lymphotropic virus type 1-associated myelopathy (HAM) [4], and CNS lymphoma [5]. We previously reported that CSF neopterin levels are elevated in patients with rheumatic meningitis and decrease after treatment [6], and we also demonstrated that measurement of the CSF neopterin level may be useful for differential diagnosis and assessment of disease activity in CNS demyelinating diseases [7]. Nevertheless, studies in larger and more diverse patient populations are needed to validate CSF neopterin as a valuable biomarker in CNS diseases.

While cut-off values for CSF neopterin have been proposed and validated for the differentiation of inflammatory and non-inflammatory diseases of the CNS with high sensitivity and specificity in neuropediatric patients [8,9], such values in non-pediatric patients have not been established. In addition, neopterin levels in adult controls vary widely, from 5 to 50 nmol/L in the CSF and from 5 to 8 nmol/L in the serum or plasma [10]. To establish the validity of CSF neopterin as a biomarker to assess neuroinflammation in adult patients with neurological disease, it should also be evaluated in a large control group.

In this study, we aimed to evaluate the CSF neopterin level in patients, divided into disease groups, including controls, to examine its usefulness as a biomarker of neuroinflammation in various CNS diseases. In addition, the effect of aging on CSF parameters, including the CSF neopterin level, was examined, as CSF protein and interleukin 6 levels, which are well-known biomarkers of neuroinflammation, reportedly increase with age [11,12]. We discovered that CSF neopterin levels were elevated in patients with various CNS diseases, reflecting CNS inflammation; they were also elevated with age.

## 2. Materials and Methods

### 2.1. Study Design and Participants

We retrospectively reviewed the medical records of patients who underwent CSF examination at Ehime University Hospital from 2005 to 2020, and patients whose CSF neopterin level was measured in our laboratory were included in this study. Diagnoses were determined from the medical records. The exclusion criteria were as follows: (i) samples collected from patients with missing information regarding age, sex, CSF cell count, or CSF protein; (ii) samples with inadequate collection or storage conditions, such as traumatic puncture; and (iii) samples collected from patients after they had undergone immunotherapy.

In this study, the following patients were classified as the control group: (i) patients diagnosed as neurologically intact; (ii) patients diagnosed with functional neurological disorders; (iii) patients diagnosed with spinal stenosis; and (iv) patients diagnosed with peripheral neuropathy not involving the CNS. Additionally, disease groups consisting of fewer than three patients were excluded from the study.

This study was approved by the Institutional Review Board for Clinical Research Ethics of Ehime University (Approval code: 2108022; Approval date: 20 August 2021) and was conducted in accordance with the Declaration of Helsinki. Written informed consent was received for the routine clinical care for all patients, and the need for additional consent for this study was waived owing to the retrospective nature of this study.

### 2.2. Measurement of Neopterin

The collected CSF samples were centrifuged at 1000× *g* for 10 min at 4 °C and stored at –80 °C until analysis in our laboratory at Ehime University. Neopterin-level analysis in CSF samples was conducted using high-performance liquid chromatography (HPLC) with fluorometric detection. The 100 µL CSF samples were thawed and immediately acidified with 100 μL of ice-cold 0.1M HCl. Subsequently, a solution comprising 1% I_2_ and 2% KI in 50 μL of 0.1M HCl was added and the reaction mixture was incubated at 20 °C in the dark for 60 min. The reaction was neutralized by adding 50 μL of 1.5% ascorbic acid, followed by centrifugation at 1000× *g* for 1 min. A 10 μL aliquot of the supernatant was injected into a C18 column (150 mm × 2.1 mm; EICOMPAK SC-5ODS, Eicom, Kyoto, Japan) for HPLC with a mobile phase comprising 3.5% methanol in water. For fluorometric detection (FP-2025 Plus, JASCO Co., Tokyo, Japan), we used an excitation wavelength of 350 nm and an emission cut-off below 410 nm.

### 2.3. Statistical Analysis

The two independent groups were compared using the chi-squared test for categorical data and Student’s *t*-test for continuous data. Spearman’s rank correlation test was used to identify associations between two variables. Multiple factor analysis was performed using stepwise logistic regression analysis and multiple regression analysis. A receiver operating characteristic (ROC) analysis was performed to examine the usefulness of the CSF neopterin level for disease differentiation. The area under the ROC curve (AUC) was calculated and the Youden index was estimated to determine the optimal cut-off value. Statistical significance was defined as a two-tailed *p*-value < 0.05. All statistical analyses were conducted using R Statistical Software (version 4.3.2: The R Foundation for Statistical Computing, Vienna, Austria).

## 3. Results

**Figure 1:**
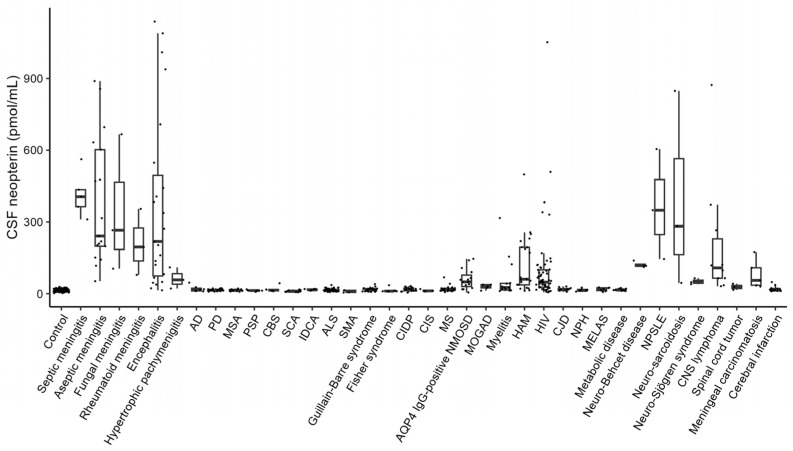
CSF neopterin level in each disease group. The box-and-whisker plot displays the median (line in the box) and lower and upper quartiles (lower and upper borders of the box). AD, Alzheimer’s disease; ALS, amyotrophic lateral sclerosis; AQP4, aquaporin-4; CBS, corticobasal syndrome; CIDP, chronic inflammatory demyelinating polyneuropathy; CIS, clinically isolated syndrome; CJD, Creutzfeldt–Jakob disease; CNS, central nervous system; CSF, cerebrospinal fluid; HAM, human T-lymphotropic virus type 1-associated myelopathy; HIV, human immunodeficiency virus; IDCA, idiopathic cerebellar ataxia; MELAS, mitochondrial encephalomyopathy, lactic acidosis, and stroke-like episodes; MS, multiple sclerosis; MSA, multiple system atrophy; MOGAD, myelin oligodendrocyte glycoprotein antibody-associated disease; NMOSD, neuromyelitis optica spectrum disorder; NPH, normal pressure hydrocephalus; NPSLE, neuropsychiatric systemic lupus erythematosus; PD, Parkinson’s disease; PSP, progressive supranuclear palsy; SCA, spinocerebellar ataxia; SMA, spinal muscular atrophy.

In total, 652 participants were included in this study. The CSF neopterin levels in each disease group were as follows (Figure 1): 13.77 ± 5.08 (mean ± standard deviation) pmol/mL in controls (*n* = 170), 415.71 ± 94.58 pmol/mL in patients with septic meningitis (*n* = 5), 381.07 ± 267.38 pmol/mL in those with aseptic meningitis (*n* = 17), 345.89 ± 289.47 pmol/mL in those with fungal meningitis (*n* = 3), 209.92 ± 138.54 pmol/mL in those with rheumatoid meningitis (*n* = 3), 366.69 ± 366.30 pmol/mL in those with encephalitis (n = 23), 63.40 ± 44.46 pmol/mL in those with hypertrophic pachymeningitis (*n* = 3), 20.25 ± 13.04 pmol/mL in those with Alzheimer’s disease (*n* = 7), 15.38 ± 3.91 pmol/mL in those with Parkinson’s disease (*n* = 27), 14.44 ± 3.81 pmol/mL in those with multiple system atrophy (*n* = 15), 13.33 ± 2.90 pmol/mL in those with progressive supranuclear palsy (*n* = 8), 18.94 ± 12.67 pmol/mL in those with corticobasal syndrome (n = 6), 10.72 ± 3.96 pmol/mL in those with spinocerebellar ataxia (*n* = 11), 16.87 ± 2.91 pmol/mL in those with idiopathic cerebellar ataxia (*n* = 7), 15.54 ± 5.47 pmol/mL in those with amyotrophic lateral sclerosis (ALS; *n* = 61), 9.73 ± 5.23 pmol/mL in those with spinal muscular atrophy (*n* = 4), 17.89 ± 7.58 pmol/mL in those with Guillain-Barré syndrome (*n* = 24), 13.98 ± 8.83 pmol/mL in those with Fisher syndrome (*n* = 8), 18.39 ± 7.82 pmol/mL in those with chronic inflammatory demyelinating polyneuropathy (*n* = 19), 13.02 ± 4.54 pmol/mL in those with clinically isolated syndrome (*n* = 4), 19.48 ± 11.58 pmol/mL in those with multiple sclerosis (*n* = 30), 60.61 ± 43.14 pmol/mL in those with aquaporin-4 (AQP4)-immunoglobulin G (IgG)-positive neuromyelitis optica spectrum disorder (NMOSD; n = 15), 28.87 ± 10.80 pmol/mL in those with myelin oligodendrocyte glycoprotein antibody-associated disease (MOGAD; *n* = 5), 58.15 ± 83.12 pmol/mL in those with myelitis (n = 15), 126.94 ± 123.03 pmol/mL in those with HAM (*n* = 21), 95.27 ± 156.02 pmol/mL in those with human immunodeficiency virus (HIV; *n* = 61), 18.36 ± 6.87 pmol/mL in those with Creutzfeldt–Jakob disease (*n* = 11), 15.59 ± 5.60 pmol/mL in those with normal pressure hydrocephalus (*n* = 7), 18.52 ± 7.56 pmol/mL in those with mitochondrial encephalomyopathy, lactic acidosis, and stroke-like episodes (*n* = 7), 16.17 ± 4.30 pmol/mL in those with metabolic disease (that is, Wernicke encephalopathy and subacute combined degeneration of the spinal cord; *n* = 11), 121.61 ± 11.89 pmol/mL in those with neuro-Behçet’s disease (n = 4), 366.50 ± 230.06 pmol/mL in those with neuropsychiatric systemic lupus erythematosus (SLE; *n* = 3), 391.94 ± 412.53 pmol/mL in those with neuro-sarcoidosis (*n* = 3), 51.22 ± 12.98 pmol/mL in those with neuro-Sjögren’s syndrome (*n* = 3), 204.59 ± 258.51 pmol/mL in those with CNS lymphoma (*n* = 10), 28.52 ± 12.98 pmol/mL in those with spinal cord tumors (*n* = 3), 80.94 ± 60.75 pmol/mL in those with meningeal carcinomatosis (*n* = 5), and 19.79 ± 11.21 pmol/mL in those with cerebral infarction (*n* = 13).

**Figure 2:**
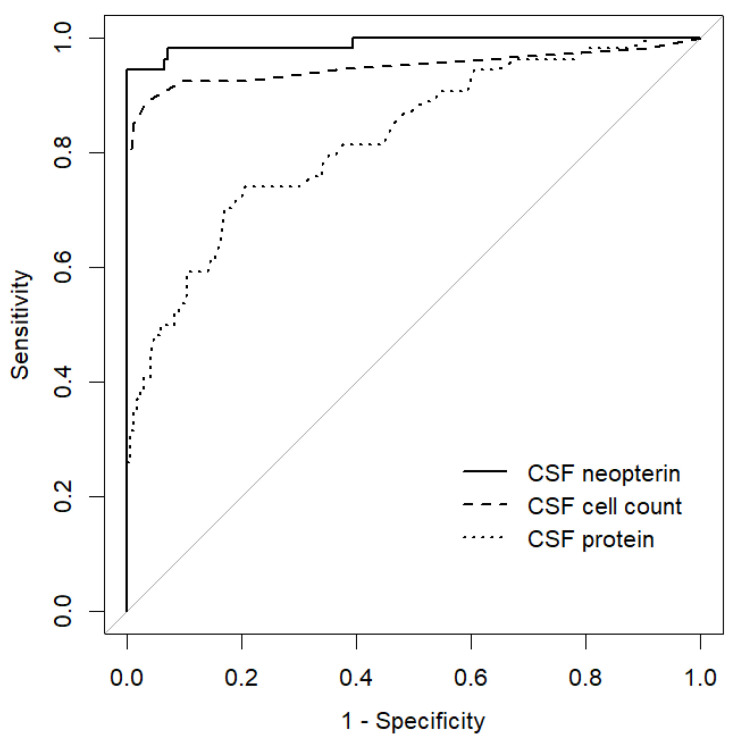
Receiver operating characteristic curves of CSF parameters (CSF neopterin, CSF cell count, and CSF protein) for discrimination of the control and meningitis/encephalitis groups. CSF, cerebrospinal fluid.

Among them, the following patient groups had mean CSF neopterin levels higher than 23.93 pmol/mL, which was the mean + 2 standard deviations (SD) of the CSF neopterin level in the control group: the septic meningitis, aseptic meningitis, fungal meningitis, rheumatoid meningitis, encephalitis, hypertrophic pachymeningitis, AQP4-IgG-positive NMOSD, MOGAD, myelitis, HAM, HIV, neuro-Behçet’s disease, neuropsychiatric SLE, neuro-sarcoidosis, neuro-Sjögren’s syndrome, CNS lymphoma, spinal cord tumor, and meningeal carcinomatosis groups. We lumped patients with septic meningitis, aseptic meningitis, fungal meningitis, rheumatoid meningitis, encephalitis, and hypertrophic pachymeningitis into the meningitis/encephalitis group (*n* = 54) as a group with CNS inflammation and examined the usefulness of CSF neopterin in differentiating these patients from the control group. Although no significant differences were observed in age (*p* = 0.899) and sex (*p* = 0.668) between the two groups, the meningitis/encephalitis group had a significantly higher CSF cell count (*p* = 0.005), CSF protein level (*p* < 0.001), and CSF neopterin level (*p* < 0.001) (Table 1). ROC analysis demonstrated that the CSF neopterin level discriminated most accurately between the meningitis/encephalitis and control groups (AUC, 0.990; optimal cut-off value, 33.57 pmol/mL; sensitivity, 100.0%; specificity, 94.4%), compared to the CSF cell count (AUC, 0.950; optimal cut-off value, 4.50/µL; sensitivity, 96.5%; specificity, 88.9%), and CSF protein level (AUC, 0.823; optimal cut-off value, 64.50 mg/dL; sensitivity, 79.0%; specificity, 74.1%) (Figure 2).

**Figure 3:**
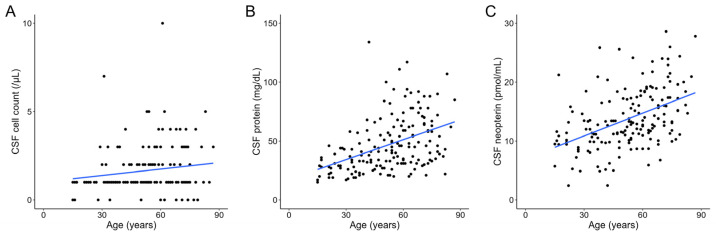
Scatter plot of age versus CSF cell count (**A**), CSF protein level (**B**), and CSF neopterin level (**C**) in the control group, fitted with a linear regression model. CSF, cerebrospinal fluid.

We examined the effect of age on CSF parameters in the control group. The control group consisted of 170 participants (99 men and 71 women) with a mean age of 52.56 ± 17.99 years. Age was significantly positively correlated with CSF protein (*r* = 0.474, *p* < 0.001) and CSF neopterin (*r* = 0.476, *p* < 0.001) levels but not with CSF cell count (*r* = 0.144, *p* = 0.061) (Figure 3). When stratified by sex, male controls were significantly (both *p* < 0.001) older (57.01 ± 15.94 vs. 46.35 ± 18.95 years) and exhibited significantly elevated CSF protein levels (54.64 ± 22.17 mg/dL vs. 36.34 ± 20.18 mg/dL) compared with female controls. No significant differences were observed in terms of CSF cell count (1.83 ± 1.39 /µL in male controls and 1.42 ± 1.32 /µL in female controls, *p* = 0.057) and CSF neopterin levels (14.08 ± 5.06 pmol/mL in male controls and 13.35 ± 5.11 pmol/mL in female controls, *p* = 0.359). Upon stepwise logistic regression analysis, only the CSF protein level was significantly associated with sex in the control group (*p* < 0.001) (Table 2). Furthermore, age exhibited significantly positive correlations with CSF protein (*r* = 0.481, *p* < 0.001) and CSF neopterin (*r* = 0.631, *p* < 0.001) levels but not with the CSF cell count (*r* = 0.056, *p* = 0.641) in female controls (Supplementary Figure S1); similarly, age exhibited significantly positive correlations with CSF protein (*r* = 0.343, *p* < 0.001) and CSF neopterin (*r* = 0.342, *p* < 0.001) levels but not with CSF cell count (*r* = 0.150, *p* = 0.140) in male controls (Supplementary Figure S2).

We also examined the above trends for the ALS group, which had the second largest number of participants (*n* = 61; 33 men and 28 women) following the control group and neopterin levels relatively close to those of the control group. With respect to clinical and laboratory profiles, the ALS group was significantly older (67.95 ± 9.85 years) than the control group (52.56 ± 17.99 years, *p* < 0.001) and the mean CSF neopterin level was significantly higher in the ALS group (15.54 ± 5.47 pmol/mL) than that in the control group (13.77 ± 5.08 pmol/mL, *p* = 0.023) (Supplementary Table S1). However, stepwise logistic regression analysis demonstrated that only age and the CSF protein level were significantly associated with group differences (*p* < 0.001 and *p* = 0.019, respectively) (Table 3).

**Figure 4:**
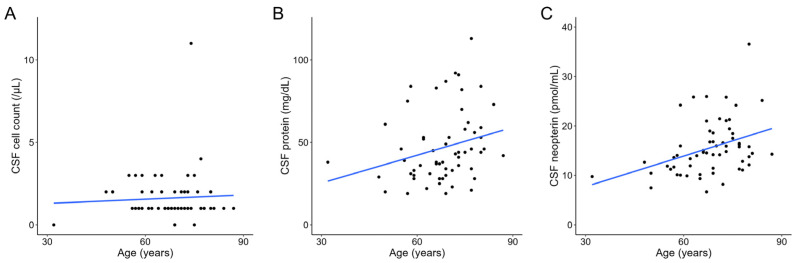
Scatter plot of age versus CSF cell count (**A**), CSF protein level (**B**), and CSF neopterin level (**C**) in patients with amyotrophic lateral sclerosis, fitted with a linear regression model. CSF, cerebrospinal fluid.

Similarly, in the ALS group, age was significantly positively correlated with CSF protein (*r* = 0.310, *p* = 0.015) and neopterin (*r* = 0.406, *p* = 0.001) levels but not with CSF cell count (*r* = −0.042, *p* = 0.746) (Figure 4). When analyzed separately by sex, age and CSF neopterin levels were significantly positively correlated in male patients (*r* = 0.593, *p* < 0.001), whereas a positive but not statistically significant correlation was observed in female patients (*r* = 0.307, *p* = 0.112) (Supplementary Figures S3 and S4). In addition, age did not statistically significantly correlate with CSF protein level or CSF cell count in either male or female patients with ALS (Supplementary Figures S3 and S4).

Finally, we examined whether the CSF neopterin level is associated with clinical parameters, such as disease duration and percentage of predicted forced vital capacity (%FVC), in patients with ALS. When patients were classified into groups with (*n* = 33) or without (*n* = 28) bulbar dysfunction, %FVC was significantly lower in the group with bulbar dysfunction, while other parameters, including the CSF neopterin level, did not significantly differ between the two groups (Table 4). In addition, multiple linear regression analysis revealed that age, but not disease duration or %FVC, was independently significantly associated with the CSF neopterin level in patients with ALS (Table 5).

## 4. Discussion

This study showed that the CSF neopterin level can be used to discriminate between patients with CNS inflammation and controls with remarkably high sensitivity and specificity. Furthermore, the CSF neopterin level exhibited a significant positive correlation with age in the control group, and this trend was also observed in patients with ALS regardless of disease duration or respiratory function.

In this study, elevated CSF neopterin levels were observed in several disease groups, as noted in the results section. Elevated levels of CSF neopterin significantly correlate with disease progression in patients with HAM [4] and may lead to a diagnosis of CNS lymphoma [13]. Most patients with HIV who are untreated have elevated levels of CSF neopterin, especially those with neuropsychiatric symptoms [14]. In terms of CNS demyelinating diseases, we recently reported that CSF neopterin levels are elevated in patients with AQP4-IgG-positive NMOSD and MOGAD compared to those in patients with multiple sclerosis [7]. In patients with neuro-Behçet’s disease and neuro-sarcoidosis, CSF neopterin levels are elevated during the exacerbation or acute phase of neurological symptoms and decreased during the remission or chronic phase of such [15]. A recent study revealed that CSF neopterin levels were significantly higher in patients with neuropsychiatric SLE than in those with SLE who lacked neuropsychiatric manifestations [16]. In addition, patients with Sjögren’s syndrome reportedly have elevated neopterin levels in the serum and saliva [17], although CSF neopterin has been reported in only one patient with Sjögren’s syndrome who presented with neuropsychiatric symptoms [18]. Furthermore, this study revealed that the CSF neopterin level can be used to differentiate the meningitis/encephalitis group from the control group with very high sensitivity and specificity compared to the CSF cell count and CSF protein level. In another study, elevated CSF neopterin levels were observed more frequently than pleocytosis in pediatric patients with inflammatory or immune-mediated diseases [19]. This study demonstrated that CSF neopterin levels are elevated in patients with a variety of neurological diseases with CNS neuroinflammation, suggesting that it is a sensitive biomarker of CNS inflammation and may be used for symptom assessment and differential diagnosis of such patients.

The upper limit (mean + 2 SD) of CSF neopterin levels in the control group was 23.93 pmol/mL in this study. In previous reports, the upper limits of the reference ranges for CSF neopterin were mainly clustered between 20 and 34 nmol/L [8]. In addition, the ROC analysis revealed that the optimal cut-off value of CSF neopterin to distinguish the control and meningitis/encephalitis groups was 33.57 pmol/mL. In our previous study, a cut-off value of 28.06 pmol/mL of CSF neopterin could discriminate between the active and inactive phases of AQP4-IgG-positive NMOSD with a sensitivity of 83.3% and specificity of 94.1% [7]. Based on the above, this study suggests that the cut-off value for CSF neopterin to identify the presence of CNS inflammation is approximately 30 pmol/mL in non-pediatric patients.

This study revealed that the CSF neopterin level increases with age. Neuroinflammation plays a significant role in age-related neurodegenerative diseases such as Alzheimer’s disease and Parkinson’s disease [20]. In healthy adults, inflammatory cytokines in the CSF increase with age [21], and age-related immune abnormalities are known to occur in the CSF [22]. CSF levels of interferon-gamma-induced protein 10, a cytokine induced by interferon-gamma like neopterin, also increase linearly with age [21], and a positive correlation between the CSF neopterin level and age has been discovered among healthy women [23]. The increase in CSF neopterin with age may reflect age-related neuroinflammation. In addition, high neopterin production is associated with an increased production of reactive oxygen species [24]. Thus, the increased oxidative stress associated with aging may affect the increase in CSF neopterin.

In the control group in this study, the CSF protein level exhibited a significant positive correlation with age and was significantly higher in men than in women. A previous study also revealed that increasing age is independently associated with higher CSF protein levels in control participants and that the CSF protein level is higher in men (57.1 ± 18.8 mg/dL) than in women (45.3 ± 15.5 mg/dL) [11]. In contrast, this study revealed no significant difference in CSF neopterin levels between male and female controls and a significant positive correlation between the CSF neopterin level and age in both sexes. Thus, we suggest that neopterin levels in the CSF increase with age, regardless of sex.

Previous studies in adult controls have revealed a mean CSF neopterin level of 16.0 ± 5.3 nmol/L at a mean age of 55 ± 18 years (n = 39) [25] and a median CSF neopterin level of 24.7 nmol/L (19.4–30.6 nmol/L) at a median age of 82 years (interquartile range, 60–96 years; n = 73) [26]. In this study, the mean age of the control group was 52.56 ± 17.99 years and the mean CSF neopterin level was 13.77 ± 5.08 pmol/mL. These results suggest that the variation in CSF neopterin levels in controls across studies reflects discrepancies related to the age of the participants.

As in the control group, the CSF neopterin level was significantly positively correlated with age in patients with ALS. Previous studies have revealed that CSF [25] and urinary [27] neopterin is elevated in patients with ALS compared with controls. In this study, multivariable analysis demonstrated an independent difference in the CSF protein level, but not in the CSF neopterin level, between the control and ALS groups. CSF protein levels are elevated in patients with ALS compared to those in age-matched controls [28]. Furthermore, CSF neopterin levels reportedly do not differ between patients with and without bulbar dysfunction and were not associated with the %FVC, which is associated with survival and disease progression in patients with ALS [29] and disease duration. These results indicate that CSF neopterin levels increase with age in patients with ALS as well as in controls, independently of clinical symptoms.

This study was conducted retrospectively and might have included cases in which CSF neopterin levels were slightly affected by inflammation outside of the CNS. In addition, inflammatory markers other than neopterin were not evaluated in this study. The strength of this study is its comprehensive evaluation of 652 patients across 38 distinct disease groups, including 170 in the control group, thereby establishing it as the most extensive study of CSF neopterin levels reported to date.

## 5. Conclusions

In this study, CSF neopterin levels were elevated in patients with various CNS diseases. A cut-off value of CSF neopterin of approximately 30 pmol/mL may be useful in assessing the activity of CNS inflammation and differentiating between diagnoses. Furthermore, a significant positive correlation between the CSF neopterin level and age was observed in controls and patients with ALS, independently of disease duration and respiratory function. Prospective studies are needed to establish CSF neopterin as a sensitive biomarker of neuroinflammation.

## Figures and Tables

**Table 1:** Comparison of clinical and laboratory profiles between controls and patients with meningitis or encephalitis.

Parameter	Control	Meningitis/Encephalitis	*p*
*n*	170	54	
Age, years	52.56 ± 17.99	52.19 ± 21.13	0.899
Men (%)	99 (58.2)	29 (53.7)	0.668
CSF cell count, /µL	1.66 ± 1.37	420.85 ± 1925.55	0.005
CSF protein, mg/dL	46.99 ± 23.14	136.22 ± 143.59	<0.001
CSF neopterin, pmol/mL	13.77 ± 5.08	349.04 ± 297.51	<0.001

Data are shown as means ± standard deviations unless otherwise indicated. CSF, cerebrospinal fluid.

**Table 2:** Stepwise logistic regression analysis with sex in the control group.

Parameter	Estimate	Standard Error	z Value	*p*
(Intercept)	−2.255	0.575	−3.921	<0.001
Age	0.016	0.011	1.549	0.121
CSF protein	0.039	0.011	3.741	<0.001

CSF, cerebrospinal fluid.

**Table 3:** Stepwise logistic regression analysis with control vs. amyotrophic lateral sclerosis.

Parameter	Estimate	Standard Error	z Value	*p*
(Intercept)	5.070	0.902	5.623	<0.001
Age	−0.081	0.014	−5.666	<0.001
CSF protein	0.019	0.008	2.348	0.019

CSF, cerebrospinal fluid.

**Table 4:** Comparison of clinical and laboratory profiles in patients with amyotrophic lateral sclerosis, stratified by the presence or absence of bulbar dysfunction.

Parameter	Bulbar Dysfunction −	Bulbar Dysfunction +	*p*
*n*	28	33	
Age, years	65.96 ± 11.51	70.54 ± 7.79	0.070
Men (%)	17 (60.7)	16 (48.5)	0.486
Disease duration, months	21.07 ± 16.76	13.89 ± 11.06	0.050
%FVC, % (*n* = 56)	104.24 ± 20.49	73.54 ± 24.09	<0.001
CSF cell count, /µL	1.89 ± 2.04	1.39 ± 0.79	0.200
CSF protein, mg/dL	46.61 ± 20.87	46.79 ± 22.20	0.974
CSF neopterin, pmol/mL	14.49 ± 6.73	16.44 ± 4.01	0.167

Data are shown as means ± standard deviations unless otherwise indicated. CSF, cerebrospinal fluid; %FVC, percentage of predicted forced vital capacity.

**Table 5:** Multiple linear regression analysis of CSF neopterin level and other parameters in patients with amyotrophic lateral sclerosis.

Parameter	Estimate	Standard Error	t Value	*p*
(Intercept)	4.676	6.380	0.733	0.467
Age	0.202	0.077	2.623	0.012
Male sex	−0.596	1.442	−0.414	0.681
Disease duration	−0.045	0.054	−0.827	0.412
%FVC	−0.022	0.029	−0.747	0.459

CSF, cerebrospinal fluid; %FVC, percentage of predicted forced vital capacity.

## Data Availability

The data supporting the findings of this study are available upon reasonable request from the corresponding author.

## Supplementary Materials

The following supporting information can be downloaded at: https://www.mdpi.com/article/10.3390/jcm13154542/s1, Figure S1: Scatter plot of age versus CSF cell count (**A**), CSF protein level (**B**), and CSF neopterin level (**C**) in the female control group fitted with a linear regression model; Figure S2: Scatter plot of age versus CSF cell count (**A**), CSF protein level (**B**), and CSF neopterin level (**C**) in the male control group fitted with a linear regression model; Figure S3: Scatter plot of age versus CSF cell count (**A**), CSF protein level (**B**), and CSF neopterin level (**C**) in the female patients with amyotrophic lateral sclerosis fitted with a linear regression model; Figure S4: Scatter plot of age versus CSF cell count (**A**), CSF protein level (**B**), and CSF neopterin level (**C**) in the male patients with amyotrophic lateral sclerosis fitted with a linear regression model; Table S1: Comparison of clinical and laboratory profiles between controls and patients with amyotrophic lateral sclerosis.
